# Supplementary material for: Improving Public Health Policy by Comparing the Public Response during the Start of COVID-19 and Monkeypox on Twitter in Germany: A Mixed Methods Study
Source: Vaccines (Basel). 2022 Nov 22;10(12):1985. doi: 10.3390/vaccines10121985 (PMC9787903; doi:10.3390/vaccines10121985)
Supplement: Supplementary file 1 [file vaccines-10-01985-s001.zip › vaccines-2013100-supplementary.pdf]

Table S1. Topic distribution of COVID-19 and Monkeypox terms.

No.	**COVID-19**	**%**	**Monkeypox**	**%**
1	coronaflle, zahl, aktuell, sehen, stunde, viel, stark, burgenland, erkrankt, vortag, hilfe, beitrag, bislang, mio, nennen, wort, verzeichnen, krankenhuser, patienten, zusammen, mittlerweile, wochenende, schlimm, untersttzung, gar, ansteigen, heilen, tod, gleichzeitig, fehler	6.65	mal, da, ausrufen, kaum, echt, hoch, knnen, langsam, ausbreitung, endemisch, davon, folgend, lang, komplett, pocken, funktionieren, ern, stndig, sogar, notfall, stadt, freiheit, hysterisch, ende, pandemievertrag, bedrohung, wert, stecken, administrados, propagao	4.9
2	wieder, erster, pandemie, frage, toll, essen, fhren, anzahl, eben, deshalb, ansteckung, sprechen, fragen, wann, berlegen, ende, zudem, bezirk, lieb, sinn, groe, klingen, empfehlen_welt, gedanke, pflicht, untersttzen, statt, todesopfer, wnsche, gsge	4.9	affenpock, schon, impfung, brauchen, patient, industrie, bahn, verkaufen_pervers, lieben, hren, selbst, pfizer, grippe, wodarg_impf, empfehlen, gibts, vermutlich, berall, sorge, toten, impf, wodarg, zulassen, versagen, benutzen, setzen, impfdose, stiko, bertragbar, nie	10.5
3	gut, hier, helfen, dabei, liegen, lesen, geld, hamsterkufe, absagen, hart, manahmen, sogar, handy, sitzen, eng, unterwegs, nie, zustzlich, grnde, gibts, antwort, bezahlen, beachten, pause, sonst, liebes, suppe, gesundleben, definitiv, wirken	5.1	auch, noch, geben, dann, gehen, tag, schlimm, herbst, gar, frage, wissen, vielleicht, notstand, panik, fragen, dumm, wei, bedingt, verdienen, dmlich, patent, freigeben, rum, super, berhaupt, knnt, suchen, wollen, gesicht, verharmlosen	8.2
4	auch, noch, dann, machen, person, gehen, sehr, leute, mssen, wie, ja, zeigen, traurig, nachricht, einfach, verstehen, stellen, leicht, dafr, danken, coronapandemie, bekommen, raum, manahm, leider, schlecht, meinung, schnell, sicher, herr	9.9	erster, hier, fall, infektion, arzt, rufen, todesopfer, mild, kondom, tote, meinen, verkaufen, bereits, jeweils, endlich, sprechen, fhren, freuen, registrieren, hnlich, ernst_nehmen, grtelros, unsinn, pock, angriff, mai, reisen, passend, schockierter, triggern	4.6
5	weit, insgesamt, steigen, infektion, finden, situation, kreis_lippe, wissen, unbekannt, gesundheit, besttigen, mglich, seite, gesamtzahl, dagegen, ndern, solidaritt, interview, kommentar, vormittag, klinikum, appell, falschmeldung, kraft, laufend, franzsisch, publikum, tests, verlassen, warnen	4.6	gut, wie, aktuell, also, thema, liegen, schule, gefhrlich, wichtig, pandemien, direkt, virus, bislang, million, mglicherweise, laufen, verzichten, impfgegner, klinik, global, folge, hervorragend, recht, beitrag, eindmmung, plan, hinweis, mnchen, fllt, fhrt	4.5

6	nehmen, immer, bleiben, kommen, weiter, letzter_atemzug, drosseln_atem, qual_stark, stck_stck, brustkrankheit, schlagkraft_fhrt, warum, gesund, leben, kind, anstieg, ein, dazu, lage, panik, lernen, heutig, liefern, politik, offen, video, raus, beitragen, in, darunter	5.5	affenpocken, so, neu, ganz, bekommen, glauben, schauen, herr, nennen, kriegen, ehrlich, affe, nochmal, etwas, entscheidung, thread, sag, klappen_nich, alarm, homophobie, sommer, schutz, erreger, hand, vermeiden, winter, teilen, art, app, strafe	9.1
7	jetzt, so, mensch, zeit, mal, krise, doch, da, ganz, stadt, sagen, wohl, also, eigentlich, land, letzter, hren, leer, richtig, regierung, ernst, bild, problem, spielen, reden, klein, vermutlich, verantwortung, zuhause, mchten	10.4	machen, haben, erst, hiv, kontakt, schnell, leute, luft, genau, zeit, sexuell, dabei, wo, erinnern, natrlich, grtelrose, virus, wirklich, einmal, whrend, stellen, corona, bestimmt, kalt, rzte, aids, ausbreiten, nachricht, handeln, erklrt	5.5
8	woche, wirklich, welt, klinik, angst, tglich, folge, schule, vergleich, artikel, nutzen, schließen, flchtling, sendung, ca, bitten, sorgen, entscheidung, medium, million, bedeuten, lange, griff, verzgerung, vorbildlich, sinnvoll, abends, einsatz, verlieren, entscheiden	4.2	affenpocke, sehr, eigentlich, heute, mnner, kind, immer, frau, wohl, sex, bleiben_tripper, denn_volksgesundheit, anwalt_verlocken, glaubwrdigkeit_besass, misfiel_whler, mosen, steuergelder_abzuzocken, gelten_hass, gleich, denken, bevlkerung, mittlerweile, damit, klar, betreffen, zahl, fll, ziehen, unglaublich, eigen	9.1
9	schon, tag, melden, gerade, hause, bekannt, erwarten, derzeit, dort, erstickend, soldat, intrig, alt, krank, echt, meinen, zahlen, verbreiten, daheim, experte, ntig, gebude, italienisch, stoppen, husten, epidemie, voll, etwa, krieg, schwer	5.2	ja, sagen, todesfllе, bertragen, gerade, bekannt, anderer, fordern, reichen, dazu, wahrscheinlich, verbreiten, bertragung, denn, isolation, stark, mglich, bonus, alleine, arsch, eben, warnen, risiko, gott, beginnen, trocken, ndern, kennen, eu, sofortig	5.1
10	coronavirus, geben, nur, heute, haben, besttigt, damit, virus, stecken, landkreis, strafe_atemwege, drosselen_sicherheit, weiterhin, mann, covid, davon, abend, hoffen, schreiben, denken, schaffen, kurve, vielleicht, rein, gleich, test, detail, quarantne, geschichte, her	8.6	impfen, einfach, impfstoff, leben, lassen, jahr, tun, nebenwirkung, artikel, weiter, halt, letzter, zeigen, person, vorbei, absolut, zustzlich, lernen, tweet, grtelrosen, testen, wenigstens, leicht, fahrt, offiziell, chef, manchmal, abseits, desinteressiert, impfdosen	4.6
11	neu, stehen, uhr, sterben, weltweit, genesen, sozial, frau, kurz, besttigte, jahr, todesfall, gestern, lassen, behandeln, lippe, tot, abstand, zeitpunkt, hundert, intensivstation, fallzahle,	4.9	doch, wieder, nehmen, mssen, raten, homosexuell, experte, dort, weg, daran, fallen, eh, problem, schtzt, ein, influenza, kurz, sofort, regierung, verharmlosung,	4.6

	vollkommen, demokratie, freitag, verstorben, grippe, club, zweiter, schutzausrstung		unertrglich, idee, entwicklung, passen, schmerz, dadurch, variante, hll, rede, fehlen	
12	besttigen, bitte, knne, anderer, tun, krankenhaus, bisher, bringen, teilen, rund, klar, bundeskanzlerin, erstellen, gerne, sonntag, freuen, ausnutzen, gro, notwendig, vergessen, programm, bett, wenigstens, waschen, enorm, warnung, reha, organisieren, leistung, offiziell	4.6	sehen, who, covid, richtig, ausbruch, mehr, erklren, vire, bitte, bild, international_notlage, entwickeln, maske, tedros, scheinen, treffen, generaldirektor, berliner, entscheiden, bringen, offensichtlich, beenden, tagesschau, eingedmmt, haut, exponentiell, rund, fest, aufwachen, teuer	4.5
13	coronakrise, wren, luft, maske, deutsch, bereits, genau, gelten, region, danke, ort, april, endlich, bundesregierung, erst, abflle, win, aprilscherze, todesflle, fall, prsident, vorerkrankung, samstag, verbreitung, maskenauf, genug, verhindern, doof, politisch, medikament	4.4	nur, mensch, kommen, knne, nun, monat, krankheit, sterben, pocke, besttigt, lnder, stimmen, infiziert, verschieden, ansteckend, prozent, lasst, eilmeldung, dran, erhalten, whlen, nichtmal, erde, zusammenfassung, undemokratisch, zugehen, aerosoleod, affenpockenvir, staub, anstellen	5.6
14	wichtig, infizieren, tote, nun, patient, pflegen, infos, derzeit_hautnah, coronastation_erleben, pichler_krise, fast, gesamt, selbst, gemeinsam, kaum, beschrnkung, erklren, familie, fordern, anstecken, virologe, manahme, rzte, natrlich, empfehlung, polizist, ersetzen, praktisch, auswirkung, infektionen	4.8	flle, melden, weit, angst, weltweit, bisher, todesfall, gesundheit, sonst, laborbesttigt, gerne, ausbrche_epidemie, gesundheit_bag, mai_bundesamt, viel, krankenhaus, grund, glck, behaupten, juli_uhr, fllen, kaufen, lsst, beobachten, bundesstaat, hoffen, gesamtzahl, deshalb, altern, drber	4.6
15	corona, aber, whrend, thema, mitarbeiter, langsam, halten, bieten, lieber, unternehmen, mrz, aktion, kosten, warten, zeitung, kontaktverbot, trump, katastrophe, bernehmen, tv, tragen, hoffnung, erreichbar, panikmach, telefonisch, befinden, kurzarbeit, chance, zusammenhang, dankeschnn	4.5	woche, infizieren, mann, bald, notlage, leider, falsch, besttigen, medium, allein, ding, allgemein, oben, erreichen, einzig, tod, antworten, gestern, zunchst, biologisch, vllig, pltzlich, vermuten, internet, dynamik, sthr, definitiv, immunisierung, insgesamt_bermitteln, bundeslnder_kreisen	4
16	mehr, naß, gott, glauben, positiv, brauchen, schauen, testen, kreis, treffen, ausgangssperre, kontakt, jhrig, morgen, arbeit, hin, weg, entwicklung, schutz, schn, kampf, arbeiten, moment, los,	6.9	schtzen, finden, international, ansteckung, fast, lieb, bleiben, infos, bundeslnder, gleichzeitig, sicher, warum, immunsystem, studie, maskenschutz, neurotropen, medizinisch, oft, einschtzung_situation, ausbruch_fallzahle, krank, frher,	4.3

	team, scheinen, denn, thring, kopf, mnchen		stehen_affenpockenflle, aerosole, inn, genug, helfen, drfen, hetzen, vergessen	
17	flle, infiziert, information, update, deutlich, grenze, lnder, schtzen, ausbreitung, hoch, erweiterung, bevlkerung, flchendeckend, stimmen, mhlenkreis, ffentlich, wieso, gefahr, gouverneur, leitlinie, dunkelziffer, karte, versterben, marke, online, drohen, stationr, geflchtet, strategie, bekennen	4.07	total, jetzt, aber, rate_total, pandemie, stehen, nchster, welt, visit, krieg, halten, los, varioledusing, tot, zweiter, angeblich, warten, politik, offen, club, interessieren, harmlos, erzhlen, sars, cov, erfolg, erstmal, spiel, prophezeien, schlecht	5.5
